# Empathy in Youths with Conduct Disorder and Callous-Unemotional Traits

**DOI:** 10.1155/2019/9638973

**Published:** 2019-04-11

**Authors:** Annarita Milone, Luca Cerniglia, Chiara Cristofani, Emanuela Inguaggiato, Valentina Levantini, Gabriele Masi, Marinella Paciello, Francesca Simone, Pietro Muratori

**Affiliations:** ^1^IRCCS Fondazione Stella Maris, Scientific Institute of Child Neurology and Psychiatry, Calambrone, Pisa, Italy; ^2^International Telematic University Uninettuno, Rome, Italy; ^3^La Nostra Famiglia Association, Oderzo, Treviso, Italy

## Abstract

Previous studies indicated that a lack of empathy could be considered the core feature of callous-unemotional (CU) traits in children and adolescents. The present study is aimed at exploring relationships among CU traits, cognitive and emotional dimensions of empathy, emotion recognition (basic, social, and complex emotions), and history of maltreatment in a sample of youths with conduct disorder diagnosis. The sample consisted of 60 Italian male patients (age range 11-17 years, mean age 13.27 ± 1.90 years) referred to the Department of Child and Adolescent Psychiatry (Pisa, Italy). In the whole sample, the levels of CU traits were significantly negatively associated with both cognitive and emotional dimensions of empathy; in addition, the CD patients with high levels of CU traits show significantly lower levels of empathic concern compared to those with low levels of CU traits. Clinical implications of the findings are discussed.

## 1. Introduction

Empathy is a multidimensional construct whose development begins early in life. Several studies on the development of empathy, in fact, indicated that early environments assume an important role in sustaining the neurobiological underpinnings of both cognitive and affective aspects of empathy [1]. For instance, sensitive, responsive, and supportive caregiving may influence the development of typical levels of empathy [2, 3]. Although most of previous studies on the relations between early environment and empathy development have not focused on specific components (cognitive and affective) of empathy in isolation, those that have distinguished between cognitive empathy and affective empathy have indicated that sensitive parenting may sustain the development of capacities in both domains [4]. Such caregiving has been found to promote young children's proclivity to take others' perspectives and to predict increased empathic concern and perspective taking in adolescence [5]. It has likewise been shown to predict increases in children's levels of prosocial behavior toward peers [6]. Previous studies argued that eye contact with attachment figures was critical for both emotional development and for the development of social cognition, including cognitive empathy [7, 8]. In other terms, these authors argued that reduced eye contact with attachment figures might contribute to the development of a lack of empathy. The implementation of eye gaze/emotional engagement strategies represents one of the targets of some parent training to implement child's emotional engagement, parent-child interaction, and empathy development.

In this framework, also, early negative experience such as maltreatments can influence the development of empathy; however, on this topic, the findings are still unclear.

In fact, from one side, some authors emphasized the existence of a trauma-based pathway to psychopathy in adults documenting a link between early affective deficits, CU traits, and exposure to maltreatment. Other studies highlight in children exposed to maltreatment a highly faster and more accurate emotional recognition. For instance, Dadds et al. [9, 10] indicated that also early experience of maltreatment may lead to deficit in emotion recognition skills, a measure of both domains of empathy, affective empathy and cognitive empathy. These controversial data show that pathways to high CU traits are complex, involving genetic aspect and environmental factors and both can influence the characteristic features associated with the traits (e.g., Dadds et al., 2017).

Callous-unemotional (CU) traits designate a specifier for children with conduct disorder (CD) diagnosis in the DSM-5 [11]. This specifier is used in CD patients, who show persistently over 12 months, in more than one setting, two or more of these clinical characteristics: lack of remorse or guilt, lack of empathy, unconcerned about performance, and shallow or deficient affect. CU traits are considered the precursors of the affective dimension of psychopathic personality and delineate a subtype of CD youths with a severe persistent and pervasive form of antisocial behavior with specific neurological, cognitive, emotional, and social characteristics [12–15]. Previous studies revealed an increasingly complex picture of the characteristics of CU traits; however, several studies indicated that a lack of empathy could be considered the core feature of CU traits [16]. Anastassiou-Hadjicharalambous and Warden [17] revealed deficits in patients with CD but low CU traits for both affective and cognitive aspects of empathy. In contrast, patients with CD and high CU trait children showed relative competency in cognitive, but deficits in affective, aspects of empathy. This finding suggested an affective-specific plausible dissociation of affective and cognitive aspects of empathy in patients with CD and high levels of CU traits. Dadds et al. [18] found that only deficit in affective empathy is persistent in adolescence and adulthood, even if some studies showed that in boys with CD and CU, both cognitive and affective were impaired [18, 19]. Bons et al. [20] demonstrated a different empathic profile in adolescent boys and in girls with high CU traits: boys and girls showed impairment in affective empathy, but girls showed also a similar impairment pattern in cognitive empathy too. The authors concluded that their work marked that adolescents with higher levels of psychopathic traits have not been characterized by an absolute stable pattern of empathy deficit but rather a relative deficit. Except this study (see also Jones et al. [21]), empirical data on the relations between deficits in empathy's components and levels of CU traits in CD patients are scarce. To the best of our knowledge, most of studies that have investigated the relations between CU traits and lack of empathy were conducted in a community or at-risk samples rather than in clinical samples of youths with CD diagnosis, even though the lack of empathy is a criterion for the CU specifier of CD diagnosis. In order to fill in this gap in the CU-based research, the current study is aimed at exploring the empathy characteristics in a sample of Italian youths with a CD diagnosis.

In recent years, the distinction between affective empathy and cognitive empathy has been receiving growing attention by clinical and cognitive scientists. Affective empathy is the capacity of sharing emotions with someone else; it involves the anterior insular, the cingulate cortex, and the amygdala [22]. Cognitive empathy is the capacity to understand the mental state of someone else, without a reflection of the other's affective state. Cognitive empathy includes the ability of decoding and labeling emotions. Some studies show a close relation between cognitive empathy and the theory of mind (ToM) and abilities or recognizing beliefs, desires, and intentions of someone else [23, 24]. Cognitive empathy reflects the functioning of parts of the dorsolateral prefrontal cortex, posteromedial cortex, superior temporal sulcus, and temporal-parietal junction [22].

A previous remarkable study investigated the relations between CU traits and the levels of cognitive empathy and affective empathy, in a community sample of Australian children, 3 to 13 years [18]. They found that CU traits are related to lower levels of affective empathy, independently to the participants' age. Conversely, previous studies that investigated specifically the relations between CU traits and the levels of cognitive empathy and/or ToM have generally indicated that high levels of youth CU traits are not related to impairment in cognitive empathy [21, 25, 26]. To summarize, the lack of affective empathy seems to be a primary deficit associated to the levels of CU traits in youth; conversely, the cognitive empathy abilities seem to be intact in youth with high levels of CU traits. Moul et al. [27] describe this youth as “a child who understands the emotional states and thoughts of others (intact cognitive empathy), but is unmoved by this understanding (poor affective empathy)”.

Several studies investigated problems in empathy using emotion-recognition paradigms [28]. Usually, in emotion-recognition tasks, participants are presented with images of faces expressing an emotion (happiness, sadness, angry, and fear) and asked to categorize emotions. Other tasks, such as the Reading the Mind in the Eyes task [29], use images of the eye region of more nuanced emotions (playful, comforting, irritated, and bored). Importantly, all these types of tasks measure the levels of both cognitive empathy and affective empathy. Using emotion recognition tasks, Sharp and Vanwoerden [30] showed a relation between CU traits and deficits in recognition of complex emotions rather than basic emotions. Sharp and Vanwoerden [30] concluded that the deficits of CU youths in emotion recognition are related to lower levels of cognitive empathy. Other previous studies that have used emotion recognition tasks indicated that in youth with elevated levels of CU traits, both cognitive and affective were impaired [19, 31]. Overall, findings from studies with emotion recognition tasks indicated that deficits in cognitive empathy might be more pronounced and pervasive among individuals with CU traits than previously thought.

The current study focused on the relationships among CU traits, cognitive and emotional dimensions of empathy, emotional recognition (basic, social, and complex emotions), and history of maltreatment in a sample of youths with CD diagnosis. Research based on the subgrouping of children with conduct problems characterized by high versus low levels of CU traits has been particularly informative with regard to such theoretical accounts in recent years, providing compelling support for the notion that distinct deficits in empathy map onto these subgroups. Furthermore, to address the call of this special issue, we investigated how early difficulties in parent-infant relationship (history of maltreatment) might be associated to the levels of youths' empathy. Overall, we aim

1. to explore relationships among CU traits, cognitive and emotional dimensions of empathy, emotional recognition (basic, social, and complex emotions), and history of maltreatment in a sample of youths with CD diagnosis
2. to examine the difference between CD patients with low and high CU traits on the different components of empathy

## 2. Method

### 2.1. Participants

The sample consisted of 60 male patients (age range 11-17 years, mean age 13.27 ± 1.90 years) consecutively referred to the Department of Child and Adolescent Psychiatry of Scientific Institute “Stella Maris” (Pisa, Italy). All patients were diagnosed according to a systematic evaluation, including a structured clinical interview according DSM-5 criteria, the Schedule of Affective Disorders and Schizophrenia for School-Age Children—Present and Lifetime Version K-SADS-PL [28], administered by trained child psychiatrists. Inclusion criteria for the participation in the current study were (1) DSM-5 diagnosis of conduct disorder according to K-SADS-PL, (2) a total WISC-IV IQ score above 80, and (3) no psychotic status or associated neurological disorders. The current sample and samples used in our previous studies did not overlap. All subjects participated voluntarily in the study after a written informed consent was obtained from parents or legal caregivers. The entire study protocol, which includes a wide range of neuropsychological tasks and psychopathological questionnaires, was approved by the local Ethical Committee.

### 2.2. Measures

#### 2.2.1. Psychopathic Traits

The antisocial process screening [32] was used in the current study to evaluate the levels of CU traits. The APSD is a 20-item rating scale, used in this study in its combined version (APSD parent version and APSD youth version) taking the highest score of each item. The APSD items are rated on a three-point Likert scale as not at all true (0), sometimes true (1), or definitely true (2). Factorial analysis using a nonclinical sample of 1120 children and adolescents identified a subdimension of the APSD related to callous-unemotional traits (defined by 6 items). The APSD has been shown to have reasonable reliability and validity in previous studies [33]. There is substantial support for the validity of the APSD in distinguishing subgroups of antisocial youth with more severe and aggressive behavior and characteristics similar to adult psychopathy [13, 34]. Our group translated the APSD in Italian language, using the back translation method. In the current sample, the reliability of the CU subscale of the APSD is excellent (Cronbach = .86).

#### 2.2.2. Empathic Concern and Perspective Taking

The Interpersonal Reactivity Index (IRI) [35] is a self-reported questionnaire that assesses perceived individual differences in the tendency to be empathetic. In this study, we used the Italian version of IRI [36].

IRI consists of 28 Likert-type items on a response scale with five alternatives ranging from 1 (does not describe me well) to 5 (describes me very well). The scale allows a multidimensional assessment of empathy measured by two cognitive subscales (perspective taking and fantasy) and two affective subscales (empathic concern and personal distress). Previous studies demonstrated the IRI reliability, and the IRI reliability has been found to be good in the current sample (Cronbach's alpha range from 0.70 to 0.80). The IRI scales showed high convergent validity with other questionnaires used to assess empathy as Empathy Questionnaire for Children and Adolescent [37], Jefferson Scale of Empathy [38], Empathy Quotient [36, 37], and Basic Empathy Scale [33]. In this study, we use two subscales: perspective-taking (PT) and empathic concern (EC). The subscale PT evaluates the propensity to adopt the views of others, in the everyday life, and the subscale EC investigates the tendency to experience feelings of compassion and concern for people having negative experience. Therefore, the scores of each subscale range between −14 and +14 points where higher scores indicate more empathic abilities. For the study, we used the Italian version of IRI by Albiero et al. [39]; the reliability of the scale is satisfactory with a good internal consistency [39].

#### 2.2.3. Emotion Recognition

In this study, we used the child's eye test (CET) [29] adapted from the adult version “Reading the Mind in the Eyes Test” developed by Baron-Cohen et al. This test was originally developed as a sensitive measure used to evaluate the theory of mind that consists the ability to make inferences regarding others' emotions (affective or emotional ToM) or beliefs and motivations (cognitive ToM). In recent years, CET was used in literature to test the ability to recognize basic and/or complex emotions. The test includes 28 photographs of the eye region of the face and requires participants to choose which of the four words best describes what the person in the picture is thinking or feeling. Three of the four words are foil mental state terms, while the fourth is defined as “correct.” The position of the four words is randomized for each item. Written instructions were given to each participant before starting the test. In the present study, the 28 items were divided into three subcategories, conveying basic emotions (e.g., happiness and sadness corresponding to Ekman's basic emotions, demonstrated to be cross-culturally recognized from the face and proposed to rely on innately specified mechanisms; 10 items), social emotions (e.g., guilt, arrogance, admiration, and flirtatiousness, which depended on the complex social context for their specification, 9 items), and complex mental states (e.g., interest, thoughtfulness, and boredom, which have been shown to depend most critically on information signaled by the eye region of the face; 9 items). For the total score and each subscale, correct responses are summed so that higher scores indicate better emotion recognition.

#### 2.2.4. History of Maltreatment

Maltreatment scores were collected using the maltreatment index clinician-child report (MI) [40]. The MI is based on the Maltreatment Classification System by Barnett et al. and uses a 4-point Likert scale (1 = never) to rate the veracity of three statements pertaining to emotional abuse, physical abuse, and neglect. MI ratings were produced by taking the highest score in the clinician or patient report. The rate of MI rating agreement was .84 (*k* Cohen). For the statistical analyses, we combined physical and emotional maltreatment into a combined physical/emotional maltreatment (active maltreatment).

### 2.3. Statistical Analyses

With regard to the first aim, associations among measures of components of empathy, CU traits, and history of maltreatment were explored using Pearson's correlations. With regard to the second aim, the CD patient group was divided in two subgroups using the cutoff for the CU subscale as reported in the APSD manual [32]. Specifically, we divided the sample in CD youths with high levels of CU traits (≥9) and CD youth with low levels of CU traits (<9). The group differences on main study variables were analyzed by Student's *t*-test. All statistical analyses were performed using SPSS v.25.0.

## 3. Results

Descriptive statistics for all variables are presented in Table 1. The levels of CU traits were significantly negatively associated with empathic concern (*r* = −.29, *p* < .05) and negatively associated with perspective taking abilities (*r* = −.27, *p* < .05). The levels of CU traits were not associated with abilities in emotion recognition and history of maltreatment.

### 3.1. Empathic, Emotional, and Behavioral Profiles of CD Patients with High CU Traits

As shown in Table 2, the *t*-test revealed a significant difference between subjects with and without elevated levels of CU traits only on empathic concern (*t* = 2.10; *p* = .04). In particular, the CD patients with high levels of CU traits show significantly lower levels of empathic concern compared to those with low levels of CU traits.

## 4. Discussion

Empathy is a multidimensional construct that includes two distinct but interrelated abilities. Cognitive empathy is the ability to understand and identify another's affective state, while affective empathy refers to share another person feelings [18, 41, 42]. Impaired empathy is a core feature in children and adolescents with CD and high CU traits, but there is limited and controversially research-concerning individuation of specific empathy deficits in CD youths. Therefore, many authors stressed the need to analyze empathy as a psychological process involved in CD youths and to develop more specific assessment [43].

One of the aims of our work was to analyze the differences in empathic concern (an aspect of affective empathy) and perspective taking (an aspect of cognitive empathy) in a clinical sample of CD patients subgrouping in CD with or without CU traits. The findings showed a specific impairment of affective empathy in patients with CD and CU traits: they lack in the ability to experience feelings of compassion and concern for people having negative feelings. No significant difference was detected in perspective taking (an aspect of cognitive empathy) between CD youths with or without high CU traits. These findings are partially in agreement with previous research into CU traits and their correlations with impaired imbalance dimensions of empathy. In community children, Anastassiou-Hadjicharalambous and Warden [44] found that CU traits are associated with deficits in both cognitive empathy and affective empathy. Similarly to our findings, Dadds et al. [18] found that typically, development children with higher levels of CU traits had lower parent-reported affective empathy level, whereas Muñoz et al. [45] in a preadolescent community sample showed that the group highest in CU traits was lowest only in affective empathy and Seara-Cardoso et al. [46] found that the affective/interpersonal component of psychopathy is associated with weaker affective empathy in an adolescent community sample. Finally, Pardini et al. [47] found that adolescents with elevated levels of CU traits showed low levels of affective empathy while controlling for delinquency and conduct problems and Jones et al., [21] using a sample of children and adolescent boys with conduct problems, found that CD children with high levels of CU traits are impaired in affective empathy but not in cognitive empathy. In a recent work, with a sample of adults from the community, authors found that both components of empathy negatively correlated with CU traits. However, the negative correlations observed between self-reported affective empathy and CU traits are significantly larger than the correlations between CU traits and cognitive empathy [48].

Several studies indicated that CD children and adolescents with CU traits had deficits in emotion processes and in orienting to affective stimuli; furthermore, they showed low fearful inhibition and are under arousal in the sympathetic autonomic nervous system. These CU characteristics may determine deficits in affective empathy as well as in cognitive empathy. Our findings are partially in line with the well-studied subject [15]; in our sample, cognitive empathy deficits are not restricted to CD children with high-level CU traits. These findings have important clinical implications, since they suggest that CD with CU traits is primarily characterized by low levels of affective empathy.

Differences in our emotion recognition task did not mark CD patients with elevated levels of CU traits. Furthermore, in our sample, correlations between CU traits and emotional recognition impairment were not significant for basic, social, and complex emotions.

In several studies, the ability to recognize fear and sadness was individuated as uniquely an impairment in adults with psychopathy traits, which explains their tendency toward aggression [49–52], but other studies have found either no deficits or superior recognition of these emotions [45, 46, 50, 53–55]. Impairments in the recognition of fear and sadness was previously described in adolescents with high levels of CU traits, but these findings were not confirmed in other studies [53, 55, 56]. Sharp et al. [56] explain discrepant findings with a unique association between CU traits and specific deficit in recognizing complex emotions and weaker associations with basic emotion recognition. Conversely, our findings did not show correlation between the levels of CU traits and recognition of both basic and complex emotions. Moreover, the levels of basic and complex emotion recognition did not discriminate the CD youths with high levels of CU traits from those with low levels of CU traits.

Marsh and Blair [57] highlighted that methods and measures for indexing ER, typically using static images on a computer screen, may only partially tap the ER impairment or do so with variable validity and reliability. Furthermore, Schwenck et al. [58] and Dawel et al. [28] indicated that ER deficits in psychopathy are pervasive across emotions and modalities and age group but revealed that important limitations in current data were that age was confounded with the sample source and that there were insufficient studies reporting results for the affective subfactor of psychopathy.

Finally, Dadds et al. [43] showed that the simple manipulation of asking a youth to look at the eyes results in increased emotion recognition. These evidences suggest that the attention to the eye region of others might improve the emotion recognition ability also in children with high levels of CU traits. Given that, we could also postulate that the characteristics of the CET stimuli help the subjects with CU traits in recognizing emotions.

Finally, in our research, the active maltreatment, evaluated using MI, did not correlate to the measures of adolescents' empathy profile and CU traits. The experience of maltreatment in childhood clearly contributes to the development of externalizing behavioral problems, empathy components, and CU trait dimension. However, the direction of influence between maltreatment and CU traits may operate through a complex interplay of heredity and environmental factors in which high CU traits can elicit harsher parenting and maltreatment [59] as well as the result from it [9].

Our study also provides some useful clinical implications. CU traits are predictors of poor response to treatment [60]. Battagliese et al. [61] indicated several treatment models for reducing aggressive behavioral problems in children and adolescents; however, only pilot studies are aimed at reducing CU traits in children and adolescents [62–65]. These models focus on the improvement of empathic behavior in children, as well as on parenting skills.

Recently, Dadds et al. [66] tested the efficacy in reducing the levels of CU traits of a specific parent training model. Although the results of this study were promising, however, the authors suggested that intervention for CU traits will need to be strengthened involving more therapeutic sessions (in terms of dose of intervention). Furthermore, they suggested augmenting behavioral with biological intervention such as oxytocin.

One of the limits of the present study is represented by the sample size, which is small and made up of males only. In particular, the sample size could significantly influence the results, which appear to be not conclusive. Further and well-oriented studies in a large sample size are needed to confirm the results.

Moreover, it could be very interesting to include females in the sample in order to explore the dimension of empathy also in females with CD.

## Figures and Tables

**Table 1:** Descriptive statistics and correlation matrix.

	M	SD	2	3	4	5	6	7
APSD CU	7.82	2.38	-.29^∗^	-.27^∗^	-.13	-.06	-.19	.11
Empathic concern	3.23	.81	—	.37^∗∗^	.09	.07	-.03	-.08
Perspective taking	2.35	.62		—	.36^∗^	-.02	.05	-.13
CET—basic	6.16	1.57			—	.39^∗^	.03	.14
CET—social	5.41	1.46				—	.33^∗^	.13
CET—complex	5.52	1.48					—	.09
Active maltreatment	2.88	.43						—

^∗∗^Significant for *p* < .001; ^∗^significant for *p* < .05.

**Table 2:** Empathic, emotional, and behavioral profiles of the CU groups.

	Low CU (*N* = 40)		High CU (*N* = 20)	
	M	Ds	M	Ds
Empathic concern	3.38^a^	.79	2.81^b^	.77
Perspective taking	2.68	.62	2.55	.80
CET—basic	5.97	1.87	6.36	1.50
CET—social	5.15	1.48	5.00	1.90
CET—complex	5.76	1.71	5.18	1.60

Different letters indicate statistically significant differences between groups.

## Data Availability

The dataset used to support the findings of this study are available from the corresponding author upon request.
